# Assessment of Able-Bodied and Amputee Cyclists’ Aerodynamics by Computational Fluid Dynamics

**DOI:** 10.3389/fbioe.2021.644566

**Published:** 2021-03-11

**Authors:** Pedro Forte, Jorge E. Morais, Tiago M. Barbosa, Daniel A. Marinho

**Affiliations:** ^1^Department of Sports, Douro Higher Institute of Educational Sciences, Penafiel, Portugal; ^2^Department of Sports Sciences, Instituto Politécnico de Bragança, Bragança, Portugal; ^3^Research Centre in Sports, Health and Human Development, Covilhã, Portugal; ^4^Department of Sports Sciences, University of Beira Interior, Covilhã, Portugal

**Keywords:** cycling, amputee, drag, rolling resistance, CFD

## Abstract

The aim of this study was to compare the aerodynamics of able-bodied and amputee cyclists by computational fluid dynamics. The cyclists’ geometry was obtained by a 3D scanner. Three CAD models were created as able-bodied, transtibial (Tt), and transradial (Tr) amputees. Numerical simulations were conducted up to 13 m/s with increments of 1 m/s to assess drag force. The drag ranged between 0.36 and 39.25 N for the able-bodied model, 0.36–43.78 for the Tr model and 0.37–41.39 N for the Tt model. The pressure drag ranged between 0.20 and 22.94 N for the normal model, 0.21–28.61 for the Tr model and 0.23–28.02 N for the Tt model. The viscous drag ranged between 0.16 and 15.31 N for the normal model, 0.15–15.17 for the Tr model and 0.14–13.38 N for the Tt model. The rolling resistance (RR) was higher on the able-bodied (2.23 N), followed by the Tr (2.20 N) and Tt (2.17 N) models. As a conclusion, the able-bodied cyclist showed less drag, followed by the Tt and Tr models, respectively. The RR presented higher values in the able-bodied, followed by the Tr and Tt models.

## Introduction

Cycling is one the most popular time-based sports. Drag force (i.e., aerodynamic resistance) plays an important role in elite cycling performance. Several studies have been carried out over the last decade on the aerodynamics of able-bodied cyclists (Defraeye et al., 2010b; Blocken et al., 2013, Blocken et al., 2018a; Forte et al., 2020a). Cycling is also popular among body-disabled people, but research on this cohort of athletes is rather scarce. Arguably, evidence gathered in able-bodied cyclists has been applied in para-cyclists (Dyer, 2016; Dyer and Disley, 2017). However, it remains to be seen if such approach is accurate. One may argue that, for instance, the aerodynamics of able-bodied cyclists and amputee counterparts might be different.

In able-bodied cyclists, drag accounts to 90% of total resistive forces, at elite racing speeds (Martin et al., 2006). The second main resistive force in cycling is rolling resistance (RR). Thus, to excel, competitive cyclists, must minimize the resistive forces and enhance the propulsive forces (Martin et al., 1998; Candau et al., 1999). To date, at least two studies modeled amputated cyclist with prosthesis (Dyer, 2014; Childers et al., 2015). However, the main performance determinants, such as, drag contribution to the resistive forces was not assessed.

In para-cycling the classifications are split-up into five classes, according to their condition (WCi, i.e., *i* = 1, 2, 3, 4, or 5 with limitations and or amputations in lower- and upper-limbs) (Tweedy and Vanlandewijck, 2011; Liljedahl et al., 2020). The drag is dependent of the form/shape of the object and the athlete’s anthropometrics (Forte et al., 2018a). Thus, an amputee may have a smaller surface drag in comparison to an able-bodied counterpart. However, no study is found comparing the aerodynamics of able-bodied and amputee cyclists [e.g., transtibial (Tt) and/or transradial (Tr)].

It is possible to assess resistive forces based on analytical procedures, experimental testing (coasting deceleration techniques, wind tunnel testing) and numerical simulations by computer fluid dynamics (Blocken and Toparlar, 2015). The latter one enables the assessment of the total drag force and its components. Total drag force is the sum of viscous drag and pressure drag. Viscous drag is strongly dependent on the form or shape of the body or system (e.g., cyclist plus bicycle, cyclist-bicycle system); whereas, pressure drag is the balance of pressure gradient between the front and back boundaries of the body or system (Schlichting and Gersten, 2016; Forte et al., 2018a). Nevertheless, remains yet elusive what is the partial contribution of each drag component to total drag force in amputees. Moreover, as far as our understanding goes, there is no comparison of the partial contribution of each drag component between amputees and able-bodied cyclists. The gold-standard method to assess drag is the wind tunnel testing. The computational fluid dynamics (CFD) analysis presented good adherence to wind tunnel data (Defraeye et al., 2010b; Blocken and Toparlar, 2015; Forte et al., 2015; Blocken et al., 2018b). Thus, CFD is deemed as a valid and reliable technique to assess drag force. Cycling is an unsteady phenomenon. Nevertheless, steady approach (static analysis) has been shown to present a good agreement with dynamic events (Crouch et al., 2014; Griffith et al., 2019).

Rolling resistance is the product between RR coefficient, mass, and gravity. Cyclists aim to minimize RR by mass reduction (bicycle, cyclist or booth) and the use of high-pressure tires with minimal deformation on the ground (Pugh, 1974). In able-bodied cyclists RR accounts about 10% of the resistive forces above 5 m/s (Blocken and Toparlar, 2015). On asphalt surface, Pugh (1974) reported RR values of 6.9 N, and a RR coefficient of 0.0081. Conversely, Candau et al. (1999), noted RR values between 2.6 and 3.5 N, and the RR coefficient between 0.0035 and 0.0039. However, no study was found comparing RR between able-bodied and amputee cyclists.

It is possible to assess the resistive forces (drag and RR) contribution by a set of analytical procedures based on the outputs of numerical simulations (Forte et al., 2015). One single study compared the total drag and energy cost by CFD and analytical procedures on able-bodied and amputee cyclists (Forte et al., 2020c). However, no details on pressure, viscous and total drag and RR variations were reported. Moreover, there is also a lack of research assessing the drag variations on amputee cyclists.

The aim of this study was to compare the aerodynamics of able-bodied and amputee cyclists (Tt and Tr) by CFD and analytical procedures. It was hypothesized that drag is higher in able-bodied cyclist, followed-up by the Tr and Tt amputee, respectively.

## Methods

### Participant

An elite level road cyclist competing at national level was recruited for this research. The bicycle was 5 kg heavy and the cyclist had 65 kg of body mass. All procedures were in accordance to the Helsinki Declaration regarding human research and a written informed consent by the volunteered participant was obtained beforehand. The Scientific Committee of the Douro Higher Institute of Educational Sciences approved this research.

### Research Design

Computational fluid dynamics enables the assessment of the aerodynamics under highly controlled conditions. This technique shows a high adherence to data collected in wind tunnel testing (Forte et al., 2015). Knowing drag force and RR, it is possible to determine the partial contribution of viscous and pressure to total drag force, as well as, the contribution of total drag and RR to total resistance forces (Candau et al., 1999). Thus, it is possible to provide a comprehensive comparison of the aerodynamics (viscous drag, pressure drag, total drag, and RR) among able-bodied, Tr, and Tt models.

### Scanning

**FIGURE 1:**
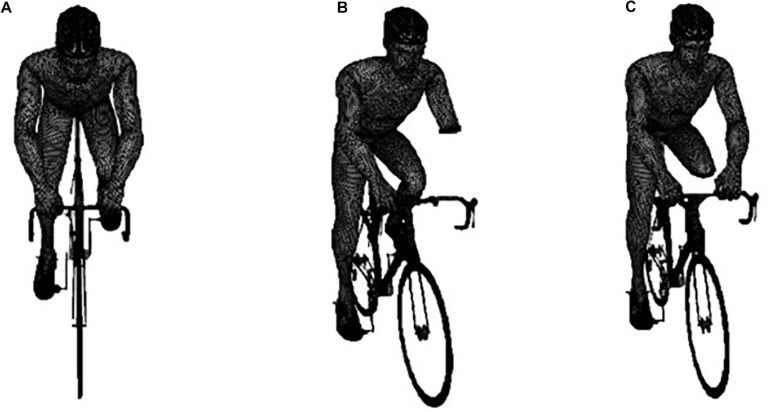
From left to right: The meshed geometry of the able-bodied **(A)**, transradial amputee **(B)**, and transtibial amputee **(C)**, on the bicycle.

The bicycle-cyclist’s geometry was collected by a Sense 3D scanner (3D Systems, Inc., Canada) and a commercially available software (Sense, 3D Systems, Inc., Canada). The cyclist was in the upright position on the bicycle (Blocken et al., 2018a). The scans were made with the participant in a static position. The geometry was edited and converted to CAD models on Geomagic Studio (3D Systems, United States) CAD models (Forte et al., 2018a). Three CAD models were created based on the single scanned participant: (Figure 1A) able-bodied (scanned); (Figure 1B) Tr amputee (edited geometry); (Figure 1C) Tt amputee (edited geometry) (Figure 1).

### Boundary Conditions

The 3D boundaries around the bicycle-cyclist system were set with 7 m of length, 2.5 m of width and 2.5 m of height on Ansys Workbench software (Ansys Fluent 16.0, Ansys Inc., Canonsburg, PA, United States). The grid, with more than 42 million of elements, was created around the geometry placed at 2.5 m distance of the fluid flow inlet portion (Blocken et al., 2013).

To generate the mesh, the automatic meshing was assigned in the Ansys Fluent 16.0. This option allows creating structured and unstructured meshes with good quality. Creating manually a 3D mesh with similar or even better quality is far more time-consuming and a very convoluted procedure for complex geometries (Marinho et al., 2010). The meshed quality was controlled based on the skewness, orthogonal quality, amount of elements, and Y+ wall turbulence values (Peters, 2009). Three different meshes were made: the polyhedral meshing; tetrahedron assembly meshing; and CutCell assembly meshing. For all meshes, fine relevance center was used. The CutCell method generated the mesh with best quality and this method created a highly structured grid (Ingram et al., 2003). The other available methods present a higher computation time and the convergence does not occur at times. The simulations with the different meshes were run at 11.11 m/s, a velocity that elite cyclists typically reach during a race (Forte et al., 2020e).

In the inlet portion, velocities up to 13 m/s with increments of 1 m/s were set at the inlet portion of the enclosure in the geometry opposite direction (−z direction). The turbulence intensity in numerical simulations was assumed as 1 × 10^–6^%. It was assumed that the bicycle-cyclist system had zero roughness non-slip wall and scalable wall functions were assigned.

### Numerical Simulations

The Reynolds-Averaged Navier–Stokes (RANS) equations were solved in Fluent CFD code (Ansys Fluent 16.0, Ansys Inc., Canonsburg, PA, United States) by the finite volume approach with the Realizable k-epsilon turbulence model. The RANS model was used based on previous cycling studies (Forte et al., 2020b,d). Moreover, the Realizable k-epsilon showed higher computation economy in comparison to Standard k-epsilon, RST and RNG k-epsilon models (Defraeye et al., 2010a,b; Forte et al., 2018a; Forte et al., 2020b).

The numerical simulations were run with 3D double-precision settings and non-equilibrium wall function. For pressure-velocity coupling the SIMPLE algorithm was used. The discretization schemes were defined as second for the pressure interpolation and the convection and viscous terms. The gradients were computed by the least-squares cell-based method. Pressure and momentum were defined as second order and second order upwind. The turbulent kinetic energy and dissipation rate were defined as first order upwind. The residuals convergence criteria of the flow parameters were set to 10 × 10^–6^. The residuals of the flow velocity components in the x-, y- and z-directions were analyzed during the simulations. The convergence occurred automatically by the Ansys Fluent 16.0 before 1,404 interactions, being the residual and drag values close to constant with negligible fluctuations.

### Outcomes

#### Drag Force

The CFD simulations yield the total, pressure and viscous drag force, as well as, its coefficients of drag. The drag force is given by Eq. 1:

(1) $$F_{D}=0.5\,\rho \,A\,C_{d}\,v^{2}$$

*F*_*D*_ is the drag force, *C*_*d*_ represents the drag coefficient, *v* the velocity, *A* the surface area, and ρ is the air density (1.292 kg/m^3^). *AC*_*d*_ is known as the effective surface area.

#### Rolling Resistance

The RR was computed by Eq. 2.

(2) $$R\,R=C_{R}\cdot m\cdot g$$

In Eq. 2, *C*_*R*_ is the rolling coefficient, *m* the body mass of the bicycle-cyclist system, and *g* the gravitational acceleration. The *C*_*R*_ was assumed as 0.0046 on car track asphalt surface (Pugh, 1974; Candau et al., 1999; Forte et al., 2015).

The body mass was estimated based on body segment parameter (Zatsiorsky and Seluyanov, 1983; de Leva, 1996; Adolphe et al., 2017). Thus, the cyclist with Tr amputation had a body mass of 64.28 kg and the cyclist with a Tt amputation 63.15 kg.

## Results

**FIGURE 2:**
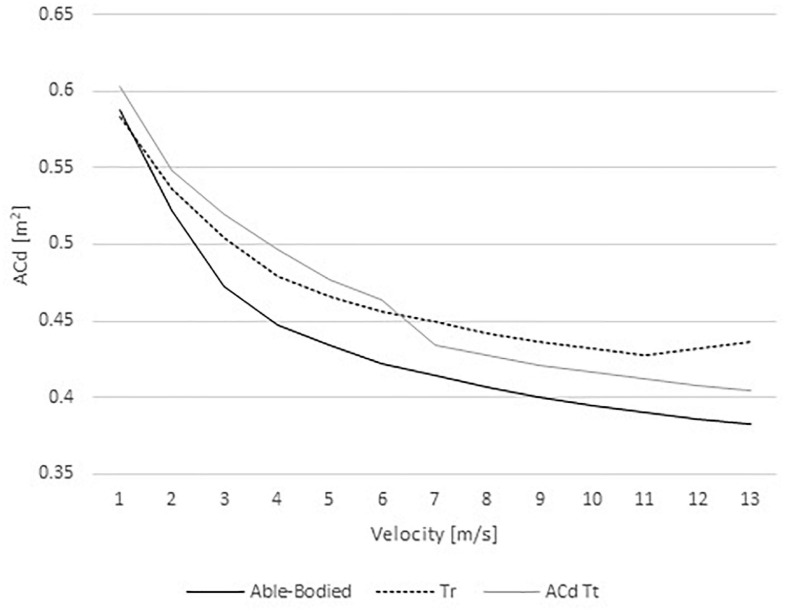
Changes of *AC*_*d*_ with increasing velocities in able-bodied cyclist, transradial (Tr), and transtibial amputees (Tt).

The *AC*_*d*_ in the able-bodied cyclist decreased with speed from 0.59 to 0.38 m^2^ (Figure 2). The Tr model *AC*_*d*_ ranged between 0.58 and 0.43 m^2^; whereas, the Tt from 0.60 to 0.40 m^2^. Hence, the able-bodied model presented an *AC*_*d*_ smaller than both amputees. Between 1 and 6 m/s, the Tr cyclist showed a smaller *AC*_*d*_ in comparison to the Tt. However, at faster velocities (from 7 to 13 m/s) the Tt was under less drag in comparison to the Tr counterpart.

**FIGURE 3:**
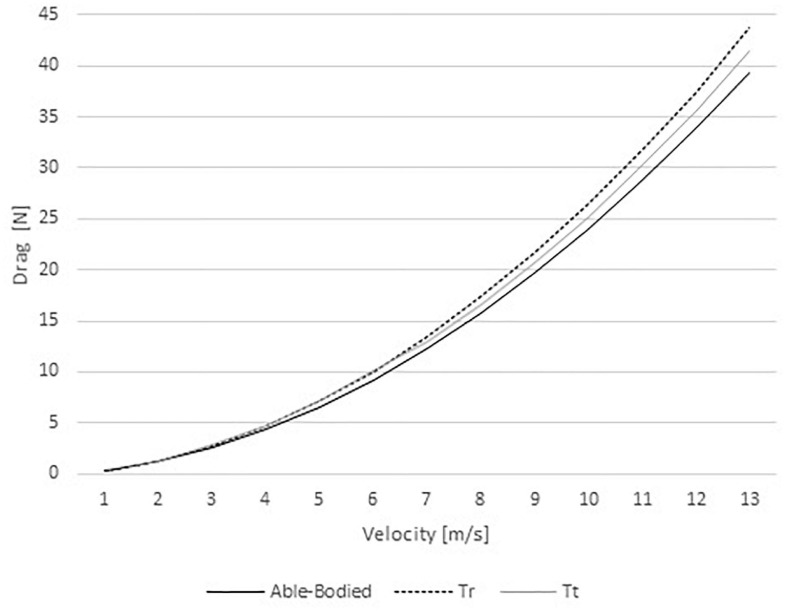
Drag variations for able-bodied, transradial (Tr), and transtibial amputees (Tt) at the selected velocities.

The drag ranged between 0.36 and 39.25 N in the able-bodied model, 0.36 and 43.78 in the Tr model, and 0.37 and 41.39 N in the Tt model (Figure 3). Therefore, the able-bodied cyclist was under less drag than the other two cyclists. Comparing both amputees, at slower velocities (1–6 m/s) the Tt model was under more drag than Tr. Conversely, at faster speeds (7–12 m/s) the Tr model was submitted to more than Tt.

**FIGURE 4:**
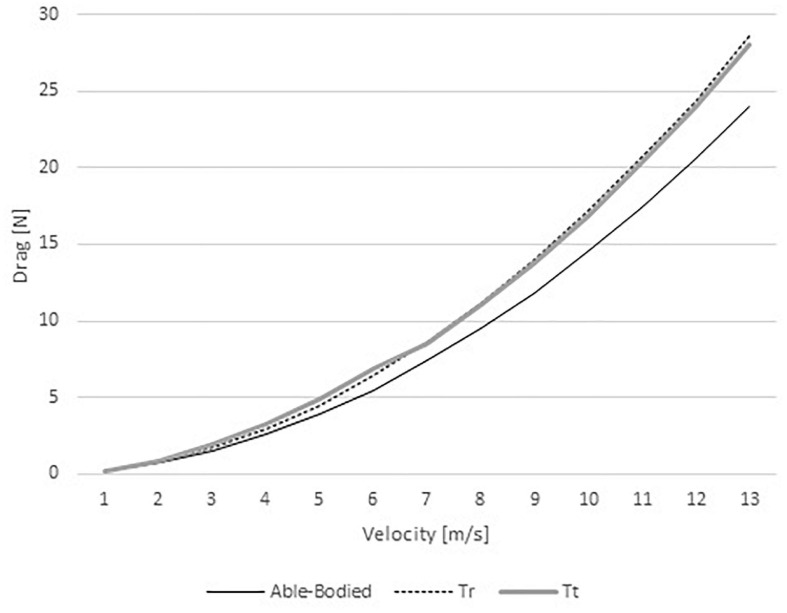
Pressure drag variation for able-bodied, transradial (Tr), and transtibial amputees (Tt) at the selected velocities.

The pressure drag ranged between 0.20 and 22.94 N in the able-bodied model, 0.21 and 28.61 in the Tr model, and 0.23 and 28.02 N in the Tt model (Figure 4). As such, one can conclude that the able-bodied cyclist was under less pressure drag than the two amputees. Again, at slow velocities (from 1 to 6 m/s) the Tt was under a larger drag force in comparison to the Tr counterpart. As abovementioned, at high speeds (7–12 m/s) the Tr cyclist presented higher drag than Tt.

**FIGURE 5:**
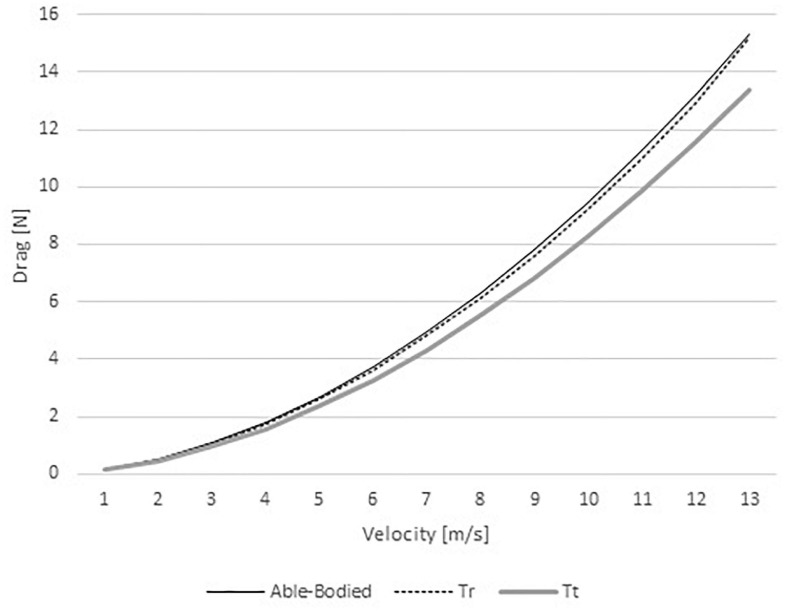
Viscous drag variation for able-bodied, transradial (Tr), and transtibial amputees (Tt) at the selected velocities.

The viscous drag ranged between 0.16 and 15.31 N in the able-bodied model, 0.15 and 15.17 in the Tr model, and 0.14 and 13.38 N in the Tt model (Figure 5). The able-bodied model showed the largest viscous drag, followed-up by the Tr and Tt model, respectively.

**FIGURE 6:**
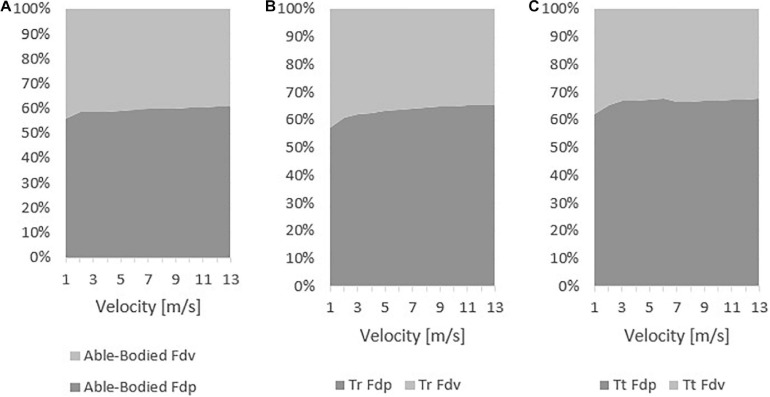
Viscous (Fdv) and pressure drag (Fdp) force contribution to total drag on able-bodied **(A)**, transradial **(B)**, and transtibial **(C)** at the selected velocities.

The viscous and pressure drag contribution to total drag for able-bodied, Tr and Tt models are depicted in Figure 6. The able-bodied viscous drag contribution to total drag ranged between 39 and 41% and decreased with velocity. The pressure drag contribution ranged from 59 to 61% and increased with velocity. The Tr viscous drag ranged between 35 and 43%, decreasing with velocity; whereas, pressure drag increased with velocity from 57 to 65%. On the Tt, viscous drag varied from 32 to 38%, decreasing with velocity. The Tt pressure drag increased with speed between 62 and 68%.

**FIGURE 7:**
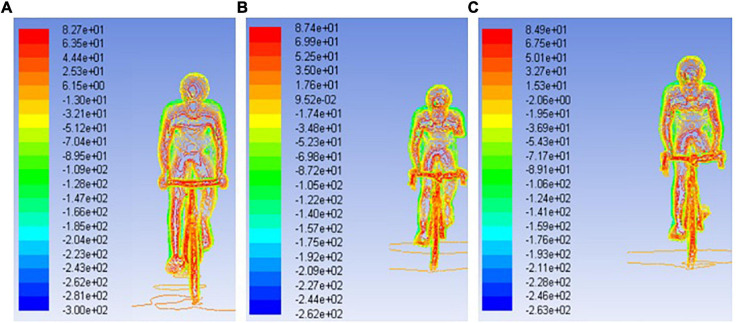
Pressure maps for able for able-bodied **(A)**, transradial **(B)**, and transtibial **(C)** at 11 m/s.

Figure 7 presents the pressure maps for the able-bodied, Tr and Tt. The able-bodied model presented the high pressure 8.27 × 10^1^ Pa, followed by the Tt with 8.49 × 10^1^ Pa and the Tr with 8.74 × 10^1^ Pa. Moreover, the lowest pressure zones were noted on the able-bodied (−3.00 × 10^2^ Pa), followed-up by the Tt (−2.63 × 10^2^ Pa) and Tr (−2.62 × 10^2^ Pa).

**FIGURE 8:**
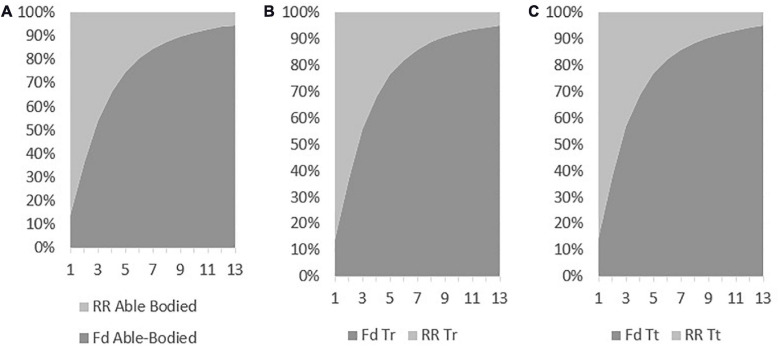
Drag and rolling resistance contribution for total resistance for able-bodied **(A)**, transradial **(B)**, and transtibial **(C)** at the selected velocities.

Rolling resistance was 2.23 N in the able-bodied, 2.20 N in the Tr, and 2.17 in the Tt (Figure 8). The difference between able-bodied and Tr was 1%. Between the able-bodied and the Tt was 3%, and between Tr and Tt was 2%. The able-bodied presented the largest RR followed-up by the Tr and Tt model, respectively. In the three models, it is possible to note that drag represents more than 50% of the resistance at speeds over 3 m/s.

## Discussion

The aim of this study was to assess the aerodynamics of able-bodied and amputee cyclists. It was hypothesized that drag is higher in able-bodied cyclist, followed-up by the Tr and Tt amputees, respectively. However, the hypothesis was rejected. The main findings of this study were that the model Tr was under the largest drag force in comparison to the Tt model and able-bodied at faster speeds (i.e., over 6 m/s). The able-bodied model showed the lowest drag force, in comparison to the selected amputee models.

In the present study, the drag was assessed by CFD. CFD was reported as valid and reliable in comparison to wind tunnel, with differences between 7 and 11% (Defraeye et al., 2010b). This methodology yields outputs on total drag, pressure and viscous drag components and its coefficients (Forte et al., 2018a, Forte et al., 2020a,b,c). It is also possible to estimate the energy cost of transportation of able-bodied and amputated cyclists from numerical simulations outputs (Forte et al., 2020c). The CFD data is underestimated by 18% in comparison to experimental techniques. The RR was assessed by an analytical procedures, having estimated the body mass based on body segment parameter (Zatsiorsky and Seluyanov, 1983; de Leva, 1996; Adolphe et al., 2017) and RR coefficient assumed to be 0.0046 on car track asphalt surface as reported by others (Pugh, 1974; Candau et al., 1999; Forte et al., 2015).

The *AC*_*d*_ in the able-bodied cyclist decreased from 0.59 to 0.38 m^2^ with increasing velocity. The Tr model *AC*_*d*_ varied between 0.58 and 0.43 m^2^; whereas, the Tt from 0.60 to 0.40 m^2^. *AC*_*d*_ values are in accordance to literature for velocities faster than 5 m/s (0.37 m^2^ ≤ *AC*_*d*_ ≤ 0.42 m^2^) for able-bodied cyclists (Grappe et al., 1997; Candau et al., 1999; Defraeye et al., 2010b). In other studies, the *AC*_*d*_ ranged between 0.261 and 0.332 m^2^ for able-bodied cyclists in the upright, dropped and time-trial position (Zdravkovic et al., 1996; Grappe et al., 1997; Candau et al., 1999; Defraeye et al., 2010a; Beaumont et al., 2018; Forte et al., 2020d). The *AC*_*d*_ variations are affected by Cd fluctuations at different speeds (Schlichting and Gersten, 2016; Forte et al., 2020d). It is expected that Cd varies about 30% on an able-bodied cyclist between 1 and 22 m/s (Forte et al., 2020d). That can be explained by the drag crisis phenomenon. The drag crisis consists in a drop of Cd values due the fluid flow transition from laminar to turbulent (Schlichting and Gersten, 2016; Forte et al., 2020d). Typically, in cycling this phenomenon may occur at Re between 3.21 × 10^5^ and 9.63 × 10^5^ and, speeds between 3 and 9 m/s. In cycling the drag crisis was assessed in the time trial position (Forte et al., 2020d). However, the present analysis was carried out in the upright position. Moreover, drag coefficient variations can be explained by the geometry shape/form, dimensions and the changes in the upright position might be different from the time trial position (Schlichting and Gersten, 2016; Forte et al., 2020d). The *AC*_*d*_ values also explain the drag variations across different speeds. Drag is dependent on the air density, velocity, surface area, and drag coefficient. The drag values were smaller on the able-bodied model, Tr was under more drag than Tt model, at faster speeds (i.e., over 6 m/s). However, Forte et al. (2020a), reported drag values between 19.66 and 21.98 N for an able-bodied model in the dropped position. These values are slightly below the results of the present study at the same velocity (28.67–31.71 N). Forte et al. (2020a), noted that cyclists in the dropped position have a smaller effective surface area (dropped position: 0.30 and 0.41 m^2^; upright position: 0.37 to 0.42 m^2^). Blocken et al. (2018a) reported an *AC*_*d*_ of 0.277 m^2^ in the dropped position matching 20.90 N in the same conditions of our study. This study was conducted in static position and the literature reported a good agreement between steady and unsteady analyses in cycling (Crouch et al., 2014; Griffith et al., 2019). Notwithstanding, amputation can cause body and bicycle rotations (Koutny et al., 2013). At least, in human swimming, this body rotation due to amputations was already assessed by CFD (Lecrivain et al., 2010).

Pressure drag values were lower for the able-bodied model, followed-up by the Tr and Tt models, respectively. Pressure drag is due to pressure differences between the front and back boundaries (Schlichting and Gersten, 2016). The fluid separation from the object at the back-boundary results in a low pressure zone due the object form/shape (Debraux et al., 2011; Schlichting and Gersten, 2016; Forte et al., 2020d). At least one study reported the pressure drag of a cyclist as being 12.56–16.51 N at 11.11 m/s, depending on the gear the subject was wearing (in the case the type of helmet). The pressure drag contribution to total drag was between 63 and 75% (Forte et al., 2020a). Other study but of an elite wheelchair sprinter in Athletics noted a pressure drag of 5.5 N at 6.5 m/s, accounting to 64% of total drag. In the present study at 7 m/s was 7 N, and at 11 m/s near 15–22 N. The contribution of pressure drag in the present study ranged between 58 and 68%. Therefore, the differences between the present study and literature can be explained by the position under assessment (Forte et al., 2020a), and differences in sport events (wheelchair racing vs. cycling) at different speeds (Forte et al., 2018a). In the present study, the pressure maps depicted less pressure in the able-bodied, followed-up by the Tt and then the Tr. That can be explained by the differences of pressure between back and front boundaries, added that the fluid flow turbulence may increase due the object shape (Schlichting and Gersten, 2016; Forte et al., 2020d). Moreover, the Tr and Tt amputations may affect the fluid flow turbulence, generating vorticities that ultimately are going to influence the drag (Schlichting and Gersten, 2016). This can explain the larger pressure drag acting on Tr and Tt. No study was founded sharing pressure maps in amputee cyclists. Thus, it is challenging to benchmark our finding with others. The order from highest to lowest pressure and pressure drag was the same. That may also justify the differences in pressure drag. The highest was the Tr, then the Tt and finally the able-bodied. This can explain why able-bodied cyclist was under less drag than the other counterparts under analysis. The amputations lead to more fluid turbulence around the limbs, the body, the cyclist-bicycle system and hence, to larger pressure differences (Forte et al., 2018a; Forte et al., 2020a).

The viscous drag ranged between 0.16 and 15.31 N in the able-bodied cyclist, 0.15 and 15.17 N in the Tr model, and 0.14 and 13.38 N in the Tt model. Once more, it was not possible to find studies on cyclist’s viscous drag in the literature. Viscous drag is related to the amount of layers of fluid that are dragged by the body (Schlichting and Gersten, 2016; Forte et al., 2018a). Larger bodies (i.e., bodies with larger surfaces) are prone to be under more viscous drag (Schlichting and Gersten, 2016; Forte et al., 2018a). Reductions in surface area, for instance, adopting different body postures, can decrease viscous drag (Forte et al., 2018a). At least one study reported that viscous drag was 10.52 and 16.51 N at 11.11 m/s in cyclists wearing aero and normal road helmet, respectively (Forte et al., 2020a). As expected, the able-bodied model of the present study was under larger viscous drag because it was the one with largest surface area. The amputee models presented less surface area, explaining the differences in viscous drag (Schlichting and Gersten, 2016; Forte et al., 2018a). The viscous drag contribution to total drag varied between 32 and 42% and decreased with velocity in the different models. At slower velocities, viscous drag is the main resistive force and the result of the system’s shape/form (Forte et al., 2015; Schlichting and Gersten, 2016).

Drag is the cyclist’s main resistive force at velocities over 5 m/s (Martin et al., 2006). In our study for velocities over 5 m/s the total drag was the highest in the Tr model, then in the Tt and lastly in the able-bodied models. The drag analyses were made in static positions. At least one study reported that the differences between static positions and dynamic leg-motions affect Cd in 6% (Crouch et al., 2016). Thus, under ecological conditions (i.e., pedaling), the drag might be 6% larger than our results. However, it is not possible to design an experimental or quasi-experimental cross-over design to compare the aerodynamics of an able-bodied and amputee. CFD remains as the most controlled technique to gather insights on this matter. Moreover, the majority of the studies that assessed cyclists aerodynamics are in static positions (Defraeye et al., 2010b; Blocken et al., 2013, Blocken and Toparlar, 2015; Blocken et al., 2018b; Beaumont et al., 2018). The total drag ranged between 0.36 and 43.78 N across the different models and speeds. The literature presents cyclists drag values between 0.16 and 76.45 N at different positions (upright, dropped and time trial) and speeds (from 1 to 22 m/s) (Forte et al., 2020a,d). It is possible to find *AC*_*d*_ values between 0.261 and 0.42 m^2^ (Zdravkovic et al., 1996; Grappe et al., 1997; Candau et al., 1999; Defraeye et al., 2010a; Beaumont et al., 2018; Forte et al., 2020d). Considering this study settings, for these *AC*_*d*_ values, the drag may vary between 0.16 and 43.40 N. Altogether, the present study shows a good agreement with literature.

The pressure drag was also the highest in the Tr model, followed-up by the Tt and able-bodied model. Conversely, the viscous drag was the highest in the able-bodied model, then the Tr model and last the Tt model. The pressure drag contribution ranged between 58 and 68%. The able-bodied model presented the high pressure, followed by the Tt and the Tr. The pressure zones highest values were 8.27 × 10^1^, 8.49 × 10^1^, and 8.74 × 10^1^ Pa for the able-bodied, Tt, and Tr, respectively. We failed to find studies assessing the pressure maps (contours) of bicycle-cyclist system. Most of the studies assessed pressure coefficients and relative velocity magnitude. The pressure differences are mainly caused by the pressure differences between the system’s front and back boundaries (Forte et al., 2015; Schlichting and Gersten, 2016). The pressure differences can possibly be explained by the vorticity around the amputee’s limbs. However, there is a lack of studies assessing the aerodynamics of amputees. More research is needed to better understand this phenomenon. At faster velocities, pressure drag had the highest contribution to total drag (Forte et al., 2020a).

The RR was 2.23 N in the able-bodied, 2.20 N in the Tr and 2.17 in the Tt. RR is dependent on the bicycle-cyclists mass and the rolling friction coefficient (Forte et al., 2018b). The RR coefficient was assumed to be 0.0046 on car track asphalt surface as reported in the literature (Candau et al., 1999; Forte et al., 2018b). In our study, the able-bodied model was the one with more mass of the three. Then, it was the Tr and finally the Tt models. Hence, it was expected RR to be larger on the able-bodied model. The RR contribution to total resistance force ranged between 10 and 90%. The RR contributions decreased with increasing velocity; whereas, drag contribution increased with the velocity. Thus, there is a trade-off in the contributions of between RR and total drag to total resistance force with increasing velocity. Drag force represented more than 80% of the total resistance force at 5 m/s. At the same velocity, it is expected that drag may contribute about 90% of the resistive forces (Martin et al., 2006). This value is within the results of our study with different models. Moreover, in an elite wheelchair sprinter, at the world record speed, RR was about 40% (Forte et al., 2018b). However, wheelchair sprinters in Athletics have larger surface areas and their maximal speed is slower than cyclists (Blocken et al., 2018a; Forte et al., 2020e).

This study reported for the first time the aerodynamics of two different amputee and compared it to an able-bodied cyclist. The main findings were that viscous, pressure and total drag vary depending on the type of amputation. Viscous drag and pressure drag are mainly affected by the amputation and the latter has an influence on the fluid flow around the limbs, body and bicycle-cyclist system. Altogether, at mean velocity, drag was higher in Tr, followed-up by Tt and then the able-bodied model. Viscous drag was the highest in the able-bodied due the larger surface area. The Tr and Tt amputation diminish the surface area and, thus the viscous drag. The pressure drag was the highest in the Tr, then the Tt and lastly the able-bodied model. That can be explained by the fluid distortions and differences of pressure between the back and front boundaries in the Tr and Tt models. RR was higher in able-bodied, followed-up by the Tr and then the Tt. Differences in RR are explained by the mass differences; where, able-bodied had more mass, followed by the Tr and Tt the lightest of the three. Findings from this study can also aid cycling committees to set specific para-sport rules.

This study has the following limitations: (1) only one able-bodied cyclist was recruited; (2) this study did not assess how the use of prosthesis can affect the aerodynamics; (3) different environmental conditions such as weather conditions (e.g., air temperature) were not assessed; (4) steady analyses were run even though the good agreement with unsteady assessments.

## Conclusion

The able-bodied cyclist model was the one under less drag force, followed-up by the Tt model and then the Tr model. At faster velocities (i.e., over 6 m/s), pressure drag was larger in the Tr cyclist, followed-up by the Tt and the able-bodied counterparts, respectively. In the case of the viscous drag, the able-bodied model showed the highest values, then the Tr and finally the Tt. The RR was higher in the case of the able-bodied, being second the Tr model and third the Tt model. In summary, the aerodynamics varies according to cyclists’ classification.

## Data Availability Statement

The raw data supporting the conclusions of this article will be made available by the authors, without undue reservation.

## Ethics Statement

The Scientific Committee of the Douro Higher Institute of Educational Sciences approved this research. The patients/participants provided their written informed consent to participate in this study.

## Author Contributions

PF, JM, and DM conceived and designed the experiments. JM and PF performed the experiments. PF and TB analyzed the data. PF and JM drafted the manuscript. TB and DM revised the manuscript. All authors contributed to the article and approved the submitted version.

## Conflict of Interest

The authors declare that the research was conducted in the absence of any commercial or financial relationships that could be construed as a potential conflict of interest.
